# Evaluation of Complete Blood Count Parameters to Predict Abnormal Uterine Bleeding

**DOI:** 10.3390/children12030327

**Published:** 2025-03-05

**Authors:** Defne Ay Tuncel, Can Acıpayam

**Affiliations:** 1 Department of Pediatric Hematology and Oncology, Adana Faculty of Medicine, Adana City Training and Research Hospital, University of Health Sciences, Adana 01370, Turkey; 2 Department of Pediatric Hematology and Oncology, Faculty of Medicine, Kahramanmaras Sutcu Imam University, Kahramanmaras 46050, Turkey; cacipayam@hotmail.com

**Keywords:** adolescents, blood cell count, child, uterine hemorrhage, hematological parameters, inflammatory markers

## Abstract

**Background/Objectives:** To investigate the diagnostic value of hematological parameters in adolescents with abnormal uterine bleeding (AUB), to make early diagnoses, and to prevent life-threatening bleeding. **Methods**: A total of 141 children, 70 patients, and 71 controls were included in the study. The laboratory findings of the patient and control groups were obtained from medical records. Cut-off values were estimated using constructing receiver operating characteristic (ROC) curves of complete blood count parameters to differentiate patients with AUB from control patients. **Results**: WBC, neutrophil, eosinophil, neutrophil/lymphocyte ratio (NLR), systemic inflammatory response index (SIRI), CRP/albumin ratio, CRP, albumin, PDW, RDW, MPV, and APTT were higher in the AUB group compared to the control group (*p* = 0.010, *p* = 0.002, *p* = 0.017, *p* = 0.017, *p* = 0.005, *p* = 0.003, and *p* < 0.001, respectively). Erythrocytes, hemoglobin, hematocrit, MCV, MCH, MCHC, fibrinogen, and INR were lower in the AUB group than in the control group (*p* < 0.001 for all). According to ROC analysis, RDW showed the highest diagnostic performance, with an AUC of 0.999 (*p* < 0.001). PDW and MPV also demonstrated high diagnostic values (AUC = 0.995 and AUC = 0.928, respectively) (*p* < 0.001). The NLR, SIRI, and CRP/albumin ratio had moderate diagnostic efficacy, with AUC values of 0.612, 0.640, and 0.642 (*p* = 0.022, *p* = 0.004, and *p* = 0.004, respectively). **Conclusions**: The high diagnostic performance of parameters such as RDW, PDW, and MPV in diagnosing AUB indicates that these parameters should be considered in clinical applications.

## 1. Introduction

Abnormal uterine bleeding (AUB) is a prevalent condition in adolescence. Uterine bleeding has etiological, structural, physiological, and idiopathic causes. Although non-structural causes are more common in adolescents, ovulation disorders and bleeding disorders are the leading physiological causes of AUB in this age group [1,2].

A multidisciplinary investigation is required to diagnose AUB. In patients referred to the hematology outpatient clinic or admitted to the emergency department, platelet dysfunctions such as immune thrombocytopenia (ITP), von Willebrand disease, liver failure, vitamin K deficiency, fibrinogen deficiency, low ferritin, etc., can be encountered. Since many patients are not diagnosed early, life-threatening bleeding occurs. Transfusion is required when Hb < 6.5 g/dL. As a result, it is essential to reduce the need for blood transfusion in patients diagnosed early, to support their psychosocial development, and to increase their quality of life [3,4].

In cases where blood transfusion is required for abnormal uterine bleeding, there may be underlying hematological disorders. Therefore, measuring the patient’s complete blood count (CBC), coagulation, von Willebrand panel, hemophilia, and factor VIII, IX, and XI levels is important. Late diagnosis can result in AUB patients receiving more blood transfusions than is necessary [5,6].

This study aimed to investigate the diagnostic value of hematological parameters in adolescents with abnormal uterine bleeding to make early diagnosis and prevent life-threatening bleeding.

## 2. Materials and Methods

A total of 141 children, 70 patients, and 71 controls were included in the study. Laboratory findings for the patient and control groups, which included routine tests such as white blood cell (WBC), erythrocyte (RBC), red blood cell distribution width (RDW), mean platelet volume (MPV), mean corpuscular volume (MCV), MCH, MCHC, neutrophil, lymphocyte, monocyte, eosinophil, basophil, hematocrit, hemoglobin, platelet, C-reactive protein (CRP), albumin, age, weight, height, and diagnosis date, were obtained using the hospital information processing system. The neutrophil/lymphocyte ratio (NLR), platelet/lymphocyte ratio (PLR), CRP/albumin ratio (CAR), systemic inflammatory response index (SIRI), systemic immune-inflammatory index (SII), and pan immune inflammation value (PIV) were calculated from these parameters. Inclusion criteria for the patient group were as follows: age between 0 and 18 years, a new diagnosis of AUB without prior treatment, and absence of any acute or chronic disease other than AUB. The control group consisted of healthy children with an age and gender profile similar to that of AUB patients. Inclusion criteria for the control group were age between 0 and 18 years, no acute or chronic disease, and absence of an active infection. Exclusion criteria were as follows: patients over the age of 18, patients with acute or chronic diseases unrelated to AUB, those who had already started treatment, and patients with missing data.

This study was approved by the University of Health Sciences, Adana Faculty of Medicine, Adana City Training and Research Hospital Ethics Committee on 15 August 2024 (decision number 102).

### Statistical Analysis

The “SPSS 17.0 for Windows” statistical package program was used to evaluate the results obtained from the experiments. Continuous variables were shown as mean ± standard deviation and categorical variables as number and percentage (*n* (%)). Variance homogeneity and suitability of variables to normal distribution were evaluated using the Levene test and the Kolmogorov–Smirnov test, respectively. In cases where the data were normally distributed and variances were homogeneous, the Student *t*-test was used for statistical analysis of the results when comparing the independent groups (patient and control groups); otherwise, the Mann–Whitney U test was used. To differentiate patients with abnormal uterine bleeding from control patients, cut-off values were estimated by constructing receiver operating characteristic (ROC) curves of complete blood count parameters, and the sensitivity and specificity values were obtained. The *p*-value was selected as 0.05 for the statistical significance assessment. The test results were considered significant if *p* < 0.05.

## 3. Results

Of the 70 AUB patients included in the study, 57 (81.4%) had von Willebrand disease, 5 (7.1%) had immune thrombocytopenia, 2 (2.9%) had factor VII deficiency, 2 (2.9%) had Glanzmann thrombasthenia, 2 (2.9%) had Barnard–Soulier syndrome, 1 (2.9%) had dysfibrinogenemia, and 1 (2.9%) had factor XI deficiency. The factor VII values for patients with factor VII deficiency were 33% and 28%, respectively. The factor XI value for the patient with factor XI deficiency was 15%. While ultrasound images were normal in 65 (92.9%) patients, splenomegaly was observed in 5 (7.1%) patients. When the accompanying bleeding status was evaluated in the patient group, it was found that 32 (45.7%) patients had nosebleeds, 23 (32.9%) patients had ecchymosis on the body as a result of impact, 10 (14.3%) patients had gum bleeding, 4 (5.7%) patients had petechiae or purpura, and 1 (1.4%) patient had no accompanying bleeding. The patients’ von Willebrand factor was 38.07 ± 31.89%, ranging from 6.80% to 136.90%.

While the mean age of the control group was 16.01 ± 2.20 years, the mean age of the AUB group was 17.79 ± 1.63 years, and this difference was statistically significant (*p* < 0.001). No significant difference was found between the groups regarding weight and height values (*p* = 0.311 and *p* = 0.322, respectively) (Table 1).

The WBC, neutrophil, and eosinophil counts were significantly higher in the AUB group compared to the control group (*p* = 0.010, *p* = 0.002, and *p* = 0.017, respectively). No significant difference was found between the groups in terms of platelet, lymphocyte, monocyte, and basophil counts (*p* = 0.169, *p* = 0.494, *p* = 0.149, and *p* = 0.150, respectively). The erythrocyte count, hemoglobin (Hb), hematocrit (Hct), MCV, MCH, and MCHC values were significantly lower in the AUB group than in the control group (*p* < 0.001 for all). However, the PDW, RDW, and MPV values were significantly higher in the AUB group compared to the control group (*p* < 0.001, for all) (Table 1).

No difference was observed between the groups in terms of PT (*p* = 0.701). However, fibrinogen and INR values were significantly lower in the AUB group compared to the control group (*p* < 0.001, *p* = 0.002, respectively), while APTT was higher (*p* < 0.001) (Table 1).

The CRP level, albumin level, and CRP/albumin ratio were higher in the AUB group than in the control group (*p* < 0.001, *p* < 0.001, *p* = 0.003, respectively) (Table 1).

The neutrophil/lymphocyte ratio (NLR) and systemic inflammatory response index (SIRI) were significantly higher in the AUB group (*p* = 0.017 and *p* = 0.005, respectively). There was no significant difference between the groups in terms of platelet/lymphocyte ratio (PLR), systemic immune-inflammatory index (SII), and pan immune inflammation value (PIV) (*p* = 0.465, *p* = 0.478, and *p* = 0.429, respectively) (Table 1).

According to ROC analysis, RDW showed the highest diagnostic performance, with an AUC of 0.999, indicating excellent ability to differentiate AUB patients from controls, with a high sensitivity of 98.6% and specificity of 97.2% (*p* < 0.001). PDW and MPV also demonstrated high diagnostic values (AUC = 0.995 and AUC = 0.928, respectively), with a sensitivity of 98.6% and 88.4% and specificity of 98.6% and 88.7% (*p* < 0.001). WBC, neutrophils, and eosinophils had moderate diagnostic efficacy, with AUC values of 0.617, 0.652, and 0.616, respectively (*p* = 0.016, *p* = 0.002, *p* = 0.018, respectively). APTT, fibrinogen, and INR had diagnostic efficacy, with AUC values of 0.879, 0.691, and 0.653, respectively (*p* < 0.001, *p* < 0.001, *p* = 0.002, respectively). The NLR, SIRI, and CRP/albumin ratio had moderate diagnostic efficacy, with AUC values of 0.612, 0.640, and 0.642, respectively, but were still significant in discriminating between AUB and controls (*p* = 0.022, *p* = 0.004, and *p* = 0. 0.004, respectively) (Table 2).

## 4. Discussion

This study aimed to investigate the diagnostic value of hematological parameters in adolescents diagnosed with abnormal uterine bleeding (AUB). AUB is a common condition during adolescence and can be caused by structural, physiological, drug-related, and organic factors. However, ovulatory disorders and bleeding disorders are among the most common causes of AUB in adolescents [7,8,9]. Early recognition of AUB is critically important for preventing life-threatening bleeding [10].

In our study, 81.4% of AUB patients presented with von Willebrand disease. This finding emphasizes the importance of recognizing underlying hematological disorders in AUB. Von Willebrand disease is a genetic disorder typically characterized by a tendency to bleed and can be a significant cause of AUB [11]. Recognizing such conditions is an important step in the treatment of patients [12].

Our study findings highlight the importance of evaluating hematological parameters in adolescents diagnosed with abnormal uterine bleeding (AUB). The significant increase in white blood cell (WBC), neutrophil, and eosinophil counts in the AUB group compared to the control group suggests that this condition may indicate an inflammatory response and the presence of underlying hematological disorders [13]. Similarly, a study conducted by Berbiç et al. found that high WBC and neutrophil counts in AUB patients are associated with inflammatory processes [14].

Additionally, the red blood cell count, hemoglobin, hematocrit, MCV, MCH, and MCHC values in the AUB group were found to be significantly lower compared to the control group (*p* < 0.001), indicating that AUB may lead to anemia. Anemia is a common condition in adolescents and can adversely affect the overall health of a patient when it occurs alongside AUB. A study examining the relationship between anemia and AUB reported that anemic conditions worsen AUB [15].

ROC analysis results indicate that RDW has the highest diagnostic performance in distinguishing AUB patients from the control group. The elevation of RDW may increase microcirculation disorders and the risk of bleeding. PDW and MPV also show high diagnostic values, indicating that these parameters play an important role in the diagnosis of AUB. Other studies have also supported the use of RDW and MPV in diagnosing AUB and obtained similar results [16,17].

It was also observed that other parameters such as NLR, SIRI, and CRP/albumin ratio showed significant differences between the AUB and control groups. These findings support the effects of systemic inflammation on AUB. Similarly, the authors noted that inflammatory markers were elevated in AUB patients, and this should be taken into account in clinical management [18].

A broader exploration of the pathophysiological basis of these changes is warranted. In related contexts, studies have highlighted the interconnected roles of inflammation, coagulation, and vascular dysfunction in conditions involving chronic blood loss. For instance, Fulghesu et al. (2024) described the importance of nuanced laboratory and imaging evaluations in early gynecological life, emphasizing the need for a comprehensive approach to diagnosing and managing gynecological conditions in adolescents [18]. Such findings underscore the importance of integrating hematological markers with clinical and imaging data to achieve a more accurate and holistic understanding of AUB.

In addition, recent studies have explored the role of platelet indices, such as mean platelet volume (MPV) and platelet distribution width (PDW), in conditions characterized by chronic blood loss and inflammation. For example, Vagdatli et al. (2010) demonstrated that elevated MPV levels are indicative of increased platelet activation, which is commonly observed in chronic inflammatory states and conditions involving significant blood loss [19]. This aligns with our findings of elevated MPV and PDW in adolescents with AUB, suggesting that these markers may reflect both compensatory mechanisms in response to chronic blood loss and underlying inflammatory processes. These insights highlight the potential utility of platelet indices as diagnostic and prognostic markers in AUB.

Furthermore, the interplay between systemic inflammation and coagulation has been extensively studied in various bleeding disorders. For instance, Kulkarni et al. (2016) investigated the role of inflammatory markers such as the neutrophil/lymphocyte ratio (NLR) and their correlation with bleeding severity in hereditary bleeding disorders [20]. Their findings suggest that systemic inflammation may exacerbate bleeding tendencies by impairing vascular integrity and altering coagulation pathways. This is particularly relevant in the context of AUB, where chronic inflammation and hormonal dysregulation may act synergistically to worsen clinical outcomes. These findings further support the integration of inflammatory markers into the diagnostic workup of adolescents presenting with AUB.

### 4.1. Study Limitations

While the findings of this study provide valuable insights into the diagnostic utility of hematological parameters in AUB, several limitations should be acknowledged. First, the study’s retrospective design may introduce selection bias, as the data were collected from a specialized hematology setting, which may not fully represent the broader adolescent population with AUB. Second, the relatively small sample size (*n* = 70) limits the generalizability of the findings. Larger prospective studies are needed to validate these results and explore additional markers that may improve diagnostic accuracy. Third, while this study focused on hematological parameters, AUB is a multifactorial condition that often requires a multidisciplinary approach, including hormonal, structural, and imaging evaluations. Future research should aim to integrate these aspects for a more comprehensive understanding of AUB.

### 4.2. Clinical Implications

The findings of this study highlight the critical role of hematological parameters, particularly RDW, PDW, and MPV, in the diagnosis and management of AUB. These parameters, which are readily available in routine complete blood count analyses, provide a cost-effective and non-invasive approach to identifying adolescents at risk for AUB. In addition, the observed associations between inflammatory markers and AUB suggest that addressing underlying inflammation may represent a novel therapeutic strategy. By integrating hematological, clinical, and imaging data, clinicians can develop more personalized and effective treatment plans for adolescents with AUB.

In conclusion, the evaluation of hematological parameters in adolescents diagnosed with AUB is critically important for the early diagnosis and prevention of life-threatening bleeding. The high diagnostic performance of parameters such as RDW, PDW, and MPV in diagnosing AUB indicates that these parameters should be considered in clinical applications. Recognizing the underlying hematological disorders of AUB is an important step in the treatment of patients.

## Figures and Tables

**Table 1:** Comparison of demographic, hematological, and biochemical parameters between control and AUB groups.

	Control (*n* = 71)	AUB (*n* = 70)	*p*-Value
Age (years)	16.01 ± 2.20	17.79 ± 1.63	<0.001
Weight (kg)	56.76 ± 8.10	58.63 ± 9.41	0.311
Height (cm)	162.99 ± 5.80	162.14 ± 5.31	0.322
WBC (×10^3^/µL)	7.32 ± 1.09	7.92 ± 1.60	0.010 *
Neutrophils (×10^3^/µL)	4.28 ± 0.70	4.92 ± 1.28	0.002
Lymphocytes (×10^3^/µL)	2.34 ± 0.64	2.28 ± 0.72	0.494
Platelets (×10^3^/µL)	309.21 ± 80.47	282.69 ± 140.01	0.169 *
Monocytes (×10^3^/µL)	0.49 ± 0.23	0.56 ± 0.26	0.149
Eosinophils (×10^3^/µL)	0.17 ± 0.09	0.22 ± 0.19	0.017
Basophils (×10^3^/µL)	0.03 ± 0.04	0.03 ± 0.07	0.150
PT (s)	11.98 ± 1.23	12.99 ± 5.98	0.701
APTT (s)	27.87 ± 3.86	40.40 ± 8.96	<0.001
Fibrinogen (g/L)	2.59 ± 0.60	2.11 ± 0.74	<0.001
INR	1.08 ± 0.13	1.01 ± 0.16	0.002
RBC (×10^6^/µL)	4.32 ± 0.35	3.07 ± 0.76	<0.001
Hb (g/dL)	12.20 ± 0.97	8.87 ± 1.36	<0.001
Hct (%)	34.95 ± 3.69	25.94 ± 4.08	<0.001
MCV (fL)	80.58 ± 4.22	75.67 ± 8.52	<0.001
MCH (pg)	25.84 ± 2.54	22.88 ± 1.24	<0.001
MCHC (g/dL)	32.79 ± 1.79	30.92 ± 1.86	<0.001
PDW (fL)	11.42 ± 1.35	16.99 ± 1.49	<0.001
RDW (%)	12.63 ± 0.93	21.70 ± 4.38	<0.001
MPV (fL)	9.72 ± 0.54	12.04 ± 1.52	<0.001
CRP (mg/L)	2.89 ± 2.10	4.11 ± 2.14	<0.001
Albumin (g/L)	42.52 ± 2.48	44.73 ± 1.55	<0.001
CAR	0.07 ± 0.05	0.09 ± 0.05	0.003
NLR	1.99 ± 0.71	2.36 ± 1.01	0.017
PLR	141.15 ± 50.89	138.01 ± 79.35	0.465
PIV	289.43 ± 177.23	389.44 ± 378.20	0.429
SIRI	0.96 ± 0.61	1.30 ± 0.84	0.005
SII	599.81 ± 221.75	687.04 ± 481.22	0.478

Mann–Whitney U test, *; Student *t*-test, neutrophil/lymphocyte ratio (NLR), platelet/lymphocyte ratio (PLR), CRP/albumin ratio (CAR), systemic inflammatory response index (SIRI), systemic immune-inflammatory index (SII), and pan immune inflammation value (PIV).

**Table 2:** Differential diagnostic performance of hematological and biochemical markers in AUB and control groups.

Item	AUC	Cut-off	Sensitivity (%)	Specificity (%)	95%CI	*p*-Value
RDW	0.999	14.20	98.6	97.2	0.000–1.000	<0.001
PDW	0.995	13.9	98.6	98.6	0.000–1.000	<0.001
MPV	0.928	10.45	88.4	88.7	0.878–0.978	<0.001
WBC	0.617	7.64	57.1	59.2	0.524–0.711	0.016
Neutrophil	0.652	4.5	60.0	64.8	0.560–0.745	0.002
Eosinophil	0.616	0.19	60.0	50.7	0.523–0.708	0.018
APTT	0.879	30.45	82.9	73.2	0.815–0.943	<0.001
Fibrinogen	0.691	2.25	73.2	62.9	0.604–0.779	<0.001
INR	0.653	1.04	52.1	65.7	0.563–0.743	0.002
NLR	0.612	2.04	52.9	60.6	0.519–0.705	0.022
SIRI	0.640	0.96	67.1	56.3	0.549–0.732	0.004
CAR	0.642	0.08	54.3	67.6	0.551–0.733	0.004

It includes the results of receiver operating characteristic (ROC) analysis to evaluate the diagnostic performance of hematologic and biochemical parameters. ROC analysis was used to calculate the area under the curve (AUC), sensitivity, and specificity for each parameter. The ROC curves were constructed to measure the ability to distinguish between AUB and control groups, and *p* < 0.05 was accepted as the level of statistical significance. Abbreviations: RDW: red cell distribution width; PDW: platelet distribution width; MPV: mean platelet volume; WBC: white blood cell; APTT: activated partial thromboplastin time; INR: international normalized ratio; NLR: neutrophil/lymphocyte ratio; SIRI: systemic inflammatory response index; CAR: CRP/albumin ratio.

## Data Availability

The datasets generated during and/or analyzed during the current study are available from the corresponding author upon reasonable request due to the privacy and ethical reasons.

## Abbreviations

The following abbreviations are used in this manuscript:

AUB	Abnormal Uterine Bleeding
ROC	Receiver Operating Characteristic
NLR	Neutrophil/Lymphocyte Ratio
CRP	C-Reactive Protein
SIRI	Systemic Inflammatory Response Index
WBC	White Blood Cell
PDW	Platelet Distribution Width
RDW	Red Cell Distribution Width
MPV	Mean Platelet Volume
APTT	Activated Partial Thromboplastin Time
MCV	Mean Corpuscular Volume
MCH	Mean Corpuscular Hemoglobin
MCHC	Mean Corpuscular Hemoglobin Concentration
INR	International Normalized Ratio
ITP	Immune Thrombocytopenia
CBC	Complete Blood Count
RBC	Red Blood Cell
PLR	Platelet/Lymphocyte Ratio
CAR	CRP/Albumin Ratio
SII	Systemic Immune-Inflammatory Index
PIV	Pan Immune Inflammation Value
